# Robot-Assisted Sigmoidectomy in a Patient With Situs Inversus Totalis: A Case Report

**DOI:** 10.7759/cureus.98938

**Published:** 2025-12-10

**Authors:** Kodai Nagakari, Hidehito Shibasaki, Yatsuka Sahara, Yu Igata

**Affiliations:** 1 Department of Gastroenterological Surgery, Japanese Red Cross Saitama Hospital, Saitama, JPN

**Keywords:** colon cancer, minimally invasive surgery, robot-assisted surgery, sigmoidectomy, situs inversus totalis

## Abstract

Situs inversus totalis (SIT) is a rare congenital anomaly characterized by a complete mirror-image transposition of thoracic and abdominal organs. Surgery in such patients is technically challenging because of the reversed anatomy, and only a few cases of robot-assisted surgery (RS) for colon cancer in SIT have been reported. We report a rare case of robot-assisted sigmoidectomy with radical lymph node dissection for sigmoid colon cancer in a 31-year-old woman with SIT. The patient presented with abdominal pain and hematochezia, and was diagnosed with sigmoid colon cancer at a previous hospital. Further examination at our institution confirmed SIT, and a robot-assisted sigmoidectomy was successfully performed with modified port placement to accommodate the mirrored anatomy. The procedure was completed safely, and the patient was discharged without complications. RS can be a safe and effective approach for patients with anatomical anomalies such as SIT.

## Introduction

Situs inversus totalis (SIT) is a rare congenital malformation, occurring in approximately one per 10,000-20,000 adults [1], and is defined by transposition of thoracic and abdominal organs across the sagittal plane. The mirrored anatomy has a significant impact on surgical procedures, and surgery in patients with SIT is generally considered technically challenging.

In recent years, favorable surgical outcomes of robot-assisted surgery (RS) for colorectal cancer have been increasingly reported [2,3], highlighting the advantages of this approach. Nevertheless, literature describing RS for colorectal cancer in patients with SIT remains extremely limited, and, to date, only three cases of colon cancer treated with RS have been documented [4,5]. RS offers wristed instruments, tremor filtration, and stable three-dimensional visualization, which may help overcome the ergonomic limitations associated with laparoscopic surgery in patients with SIT. Herein, we report a case of sigmoid colon cancer in a patient with SIT who successfully underwent RS, representing the fourth reported case of RS for colon cancer in a patient with SIT. We also discuss the technical considerations and port placement strategy that contributed to the safe and efficient completion of the procedure.

## Case presentation

**Figure 1:**
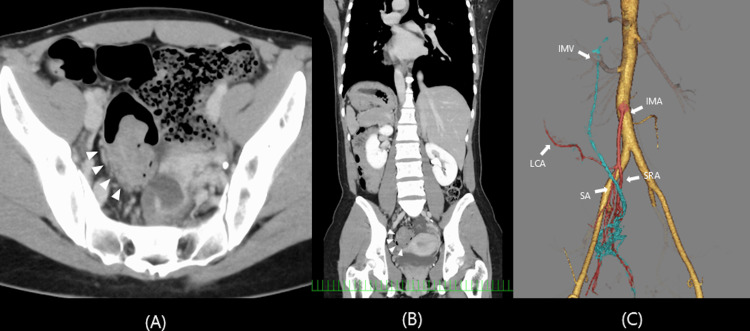
Contrast-enhanced computed tomography and 3D computed tomography angiography. Contrast-enhanced computed tomography showing a tumor in the distal sigmoid colon (arrowhead) on the axial image (A). Complete transposition of the thoracic and abdominal organs is demonstrated on the coronal image (B).  3D computed tomography angiography showed that the left colic artery and sigmoid artery had a common origin from the inferior mesenteric artery (C). IMA, inferior mesenteric artery; IMV, inferior mesenteric vein; LCA, left colic artery; SA, sigmoid artery; SRA, superior rectal artery; 3D, three-dimensional

A 31-year-old woman presented to the previous hospital with abdominal pain and hematochezia, and was diagnosed as having sigmoid colon cancer by colonoscopy. She was referred to our hospital for further examination and treatment. At our institution, colonoscopy identified a 40-mm type 2 tumor in the sigmoid colon, located 20 cm from the anal verge. Histopathological examination of the biopsy specimen confirmed well-differentiated adenocarcinoma. Contrast-enhanced computed tomography (CT) revealed SIT and localized wall thickening with contrast enhancement in the sigmoid colon. No lymph node metastasis or distant metastasis was detected. Three-dimensional CT angiography showed that the left colic artery and sigmoid artery had a common origin from the inferior mesenteric artery, which was considered a normal anatomical variation (Figure 1).

**Figure 2:**
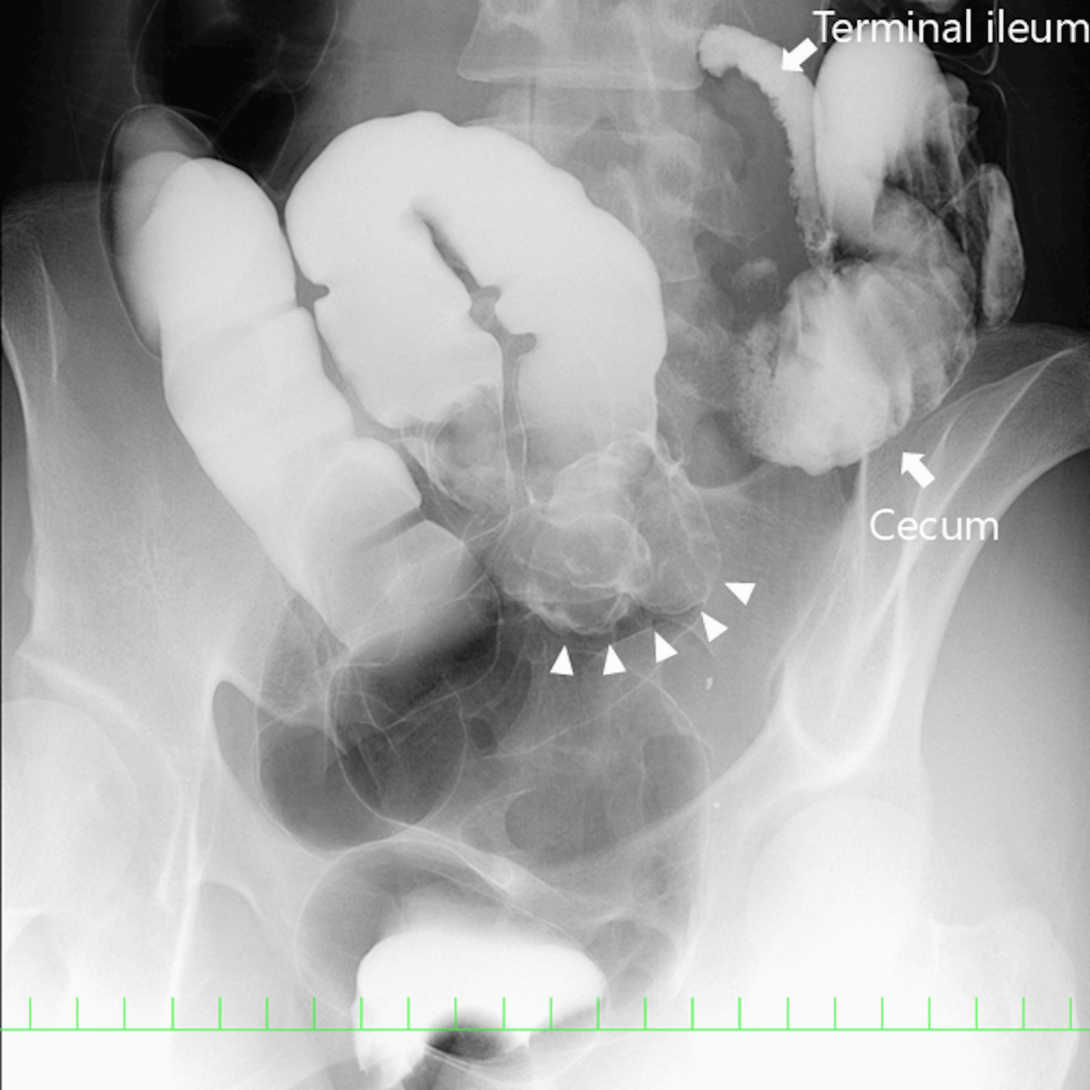
Contrast enema. Contrast enema demonstrating a tumor in the distal sigmoid colon (arrow head) in a patient with situs inversus totalis.

Contrast enema demonstrated a tumor in the distal sigmoid colon in a patient with SIT (Figure 2).

**Figure 3:**
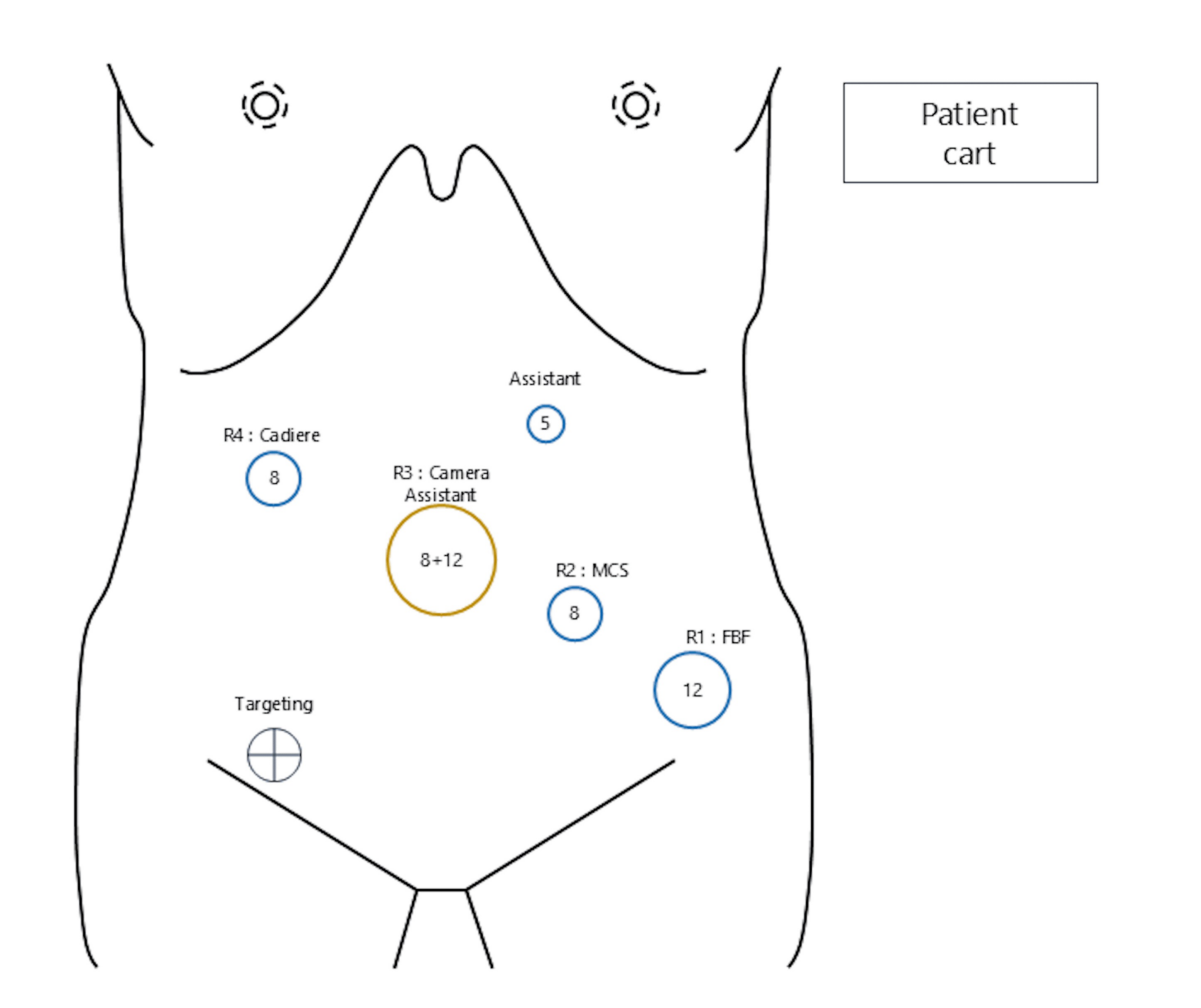
Trocar placement and arm position settings of the da Vinci surgical system Schema of trocar placement for robot-assisted sigmoidectomy in a patient with situs inversus totalis. The trocars were arranged in a mirror image of the standard configuration for left-sided colectomy. The da Vinci system was rolled in from the left caudal side of the patient. Cadiere, cadiere forceps; FBF, fenestrated bipolar forceps; MCS, monopolar curved scissors; R1-4, robotic arms

Based on these findings, a robot-assisted sigmoidectomy with radical lymph node dissection was performed. The patient was placed in the lithotomy position. The trocars were placed in the mirror image of a normal robotic sigmoidectomy setup. First, a mini-laparotomy of approximately 4 cm was made at the umbilicus, and a LapShield (LAGIS Enterprise, Taichung City, Taiwan) was inserted. A LapBase (LAGIS Enterprise) equipped with an 8-mm robotic trocar and a 12-mm assistant trocar was then attached, and pneumoperitoneum was established. An additional 12-mm robotic trocar was placed medial to the left iliac crest, an 8-mm robotic trocar was positioned at the midpoint between the umbilicus and the 12-mm robotic trocar, a 5-mm assistant trocar was inserted in the left upper abdomen, and an 8-mm robotic trocar was placed in the right upper abdomen (Figure 3). The patient was placed in a 15° Trendelenburg position with a 10° left tilt. The da Vinci Xi Surgical robot system (Intuitive Surgical, Sunnyvale, CA, USA) was docked from the left caudal side of the patient, and the robotic arms were arranged as shown in Figure 3.

**Figure 4:**
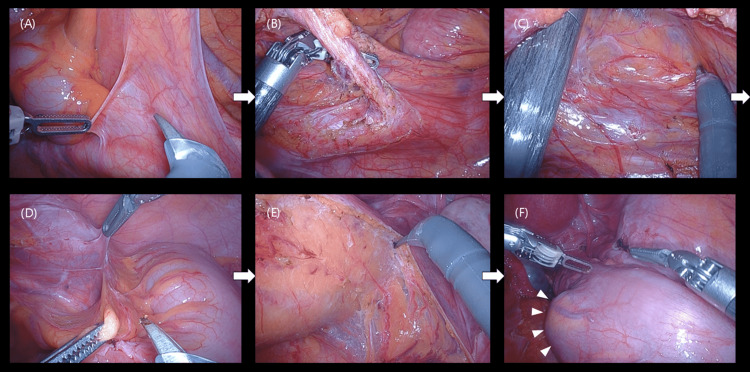
Intraoperative findings. Intraoperative view showing the ligation of the inferior mesenteric artery at its root using a medial-to-lateral approach. The mesenteric dissection and lymphadenectomy were performed safely with good visualization. (A) Peritoneal incision. (B) Ligation of the inferior mesenteric artery. (C) Mobilization of the mesocolon. (D) Lateral dissection. (E) Mobilization of the rectum. (F) Tumor location after mobilization.

During the robotic procedure, a fenestrated bipolar forceps (first arm), monopolar curved scissors and Maryland bipolar forceps (second arm), a 30° laparoscope (third arm), and Cadiere forceps (fourth arm) were used. The surgeon controlled the first arm with the left hand, while the second and fourth arms were operated by the right hand. Sigmoidectomy was performed using a medial-to-lateral approach. Inferior mesenteric artery was ligated at its root, and radical lymph node dissection was successfully completed. Reconstruction was performed using the double-stapling technique (Figure 4).

Total operation time was 204 min, console time was 139 min, and blood loss was 5 ml. The postoperative course was uneventful, and the patient was discharged on postoperative day 7. On histological examination, the tumor stage was pT3N0M0 (UICC, eighth edition [6]) with negative resection margins (PM0, 130 mm; DM0, 45 mm; RM0). All 57 retrieved lymph nodes were free of metastasis (0/57). 

## Discussion

SIT is a rare congenital malformation with an incidence of approximately one in 10,000-20,000 adults and is seldom encountered in routine clinical practice. Surgical procedures in patients with SIT are considered more challenging because surgeons are unfamiliar with the mirrored anatomy. Several reports have described surgical procedures performed in patients with SIT. Recently, case reports of RS in patients with SIT have been gradually increasing [7,8], highlighting the advantages of RS, such as precise maneuverability and superior visualization.

When discussing surgical procedures for patients with SIT, in most laparoscopic surgeries, the surgeon and assistant switch their positions to adapt to the mirror-image anatomy. Consequently, surgeons may face technical difficulties when using energy devices with their nondominant hand. If they do not change their positions, they must instead adjust to the mirrored view. However, these challenges can be overcome by RS, in which multi-jointed instruments enable surgeons to manipulate and use energy devices flexibly with their dominant hand. Furthermore, tremor filtration allows smooth and precise movements even with the nondominant hand. These advantages enhance surgical safety and facilitate complex procedures in patients with anatomical anomalies such as SIT.

To date, only seven cases of RS for colorectal cancer in patients with SIT have been reported, four of which involved rectal cancer [9-11]. Only three cases of colon cancer have been described [4,5], and the present case represents the fourth report of RS for colon cancer in a patient with SIT.

All previously reported cases utilized the da Vinci Surgical System (Xi or S). However, port placement and operative procedures varied among the reports. In most cases, more than six incisions were required, and some adopted a dual-docking approach. In contrast, our procedure was completed using only five incisions, including one 5-mm port, with a single docking. The optimal port placement was determined based on insights gained from previous reports (Table 1).

**Table 1:** Reports of robot-assisted surgery for colorectal cancer in patients with situs inversus totalis

Author	Year	Location	Complication	Blood loss	Operating time	Port placement	Docking filtration	
Leong et al. [9]	2012	Rectum	None	-	-	Square position, 6 incisions	Single docking	
Foo et al. [12]	2015	Rectum	None	100	204	Mirror position, 6 incisions	Dual docking	
Cui et al. [11]	2018	Rectum	None	50	210	Mirror position, 5 incisions	Single docking	
Kasai et al. [10]	2020	Rectum	None	Minimal	194	Straight position, 6 incisions	Dual docking	
Kato et al. [4]	2024	Ascending colon	None	5	218	Mirror position, 6 incisions	Dual docking	
Hara et al. [5]	2025	Sigmoid colon & stomach	None	70.9	535	Straight position, 5 incisions	Dual docking	
Altamirano et al. [13]	2025	Ascending colon	None	5	-	Mirror position, 5 incisions	Single docking	
Our case	2025	Sigmoid colon	None	5	204	Mirror position, 5 incisoins	Single docking	

## Conclusions

We successfully performed a robotic colectomy for sigmoid colon cancer in a patient with SIT. Mirrored port placement and thorough preoperative planning were the key to the successful outcome. The patient remains disease-free nine months after surgery. By sharing this experience, we aim to contribute to the growing evidence supporting the safety and feasibility of RS in SIT, and to provide a useful reference for surgeons encountering similar cases.
